# Medical AI Agents for Clinical Decision Support: Viewpoint Using the Planning, Action, Reflection, and Memory (PARM) Analytical Lens

**DOI:** 10.2196/92584

**Published:** 2026-07-21

**Authors:** Rasit Dinc, Nurittin Ardic

**Affiliations:** 1 INVAMED Medical Innovation Institute, One World Trade Centre, 85th Floor 285 Fulton Street, New York, NY, 10007, United States, 1 3475350630; 2 Med-International UK Health Agency Ltd., Nuneaton, Warwickshire, United Kingdom

**Keywords:** medical AI, clinical decision support, AI agents, multimodal AI, human oversight

## Abstract

Medical AI agents are emerging as a new generation of clinical decision support systems, moving beyond static prediction toward multistep, workflow-oriented assistance. This Viewpoint argues that agentic architectures incorporating planning, action, reflection, and memory (PARM) represent a meaningful evolution beyond traditional rule-based, machine learning, and multimodal clinical decision support systems. Using PARM as an analytical lens, we examine how medical AI agents can support diagnostic reasoning, treatment planning, and longitudinal monitoring while remaining constrained by human oversight. We further discuss the governance mechanisms required for responsible implementation, including bounded autonomy, auditability, verification protocols, postdeployment surveillance, and clear accountability structures. Rather than proposing autonomous modification of clinical judgment, this Viewpoint emphasizes agentic AI as a supervised workflow support paradigm. Safe implementation will require technical safeguards, institutional governance, regulatory clarity, and evaluation approaches that assess end-to-end task reliability, escalation behavior, and performance under deployment shifts.

## Introduction

Clinical decision support systems (CDSSs) have long been positioned as a cornerstone of digital medicine, aiming to improve diagnostic accuracy, treatment planning, and patient follow-up by leveraging the computational analysis of clinical data. Early CDSS implementations were largely rule-based, encoding expert knowledge into deterministic logic that operated within narrowly defined clinical domains [1]. Although effective in specific contexts, these systems often struggled to scale with the increasing complexity, heterogeneity, and volume of modern health care data. Subsequent generations of data-driven approaches, including statistical machine learning and deep learning, significantly improved predictive performance but remained fundamentally reactive, offering recommendations or risk scores without the ability to autonomously plan, act, or adapt over time [2-4].

The last decade has seen rapid advancements in multimodal AI, enabling the integration of various data types, such as clinical text, medical imaging, physiological signals, laboratory results, and increasingly, patient-generated data. Multimodal approaches have improved performance by integrating different data types [5-7]. Despite these advances, many currently reported multimodal CDSS applications remain limited to single-step inference or static estimation. They often lack the capacity for persistent memory, goal-directed behavior, and the translation of insights into coordinated clinical actions, thus limiting their impact on real-world clinical workflows. Recent advances in foundation models and tool-assisted architectures have begun to address these limitations [8]. Large language models (LLMs) and multimodal foundation models now exhibit enhanced reasoning capabilities, natural language understanding, and the ability to interact with external tools such as calculators, databases, and application programming interfaces. Frameworks integrating reasoning with action selection have shown that models can iteratively parse complex goals, call appropriate tools, and interpret results to inform further reasoning [9]. In parallel, retrieval-augmented generation has enabled dynamic access to external information sources by mitigating the limitations of static model training and improving the factual basis of decision-making in clinical contexts [10]. These developments have accelerated the emergence of medical AI agent systems designed not only to predict or advise but also to achieve clinical goals through iterative sensing, reasoning, action, and learning.

In health care settings, medical AI agents are increasingly conceptualized as autonomous or semiautonomous computational entities operating within extended clinical workflows while remaining subject to appropriate human oversight. In this study, the term “agent” refers to structured workflow support with limited autonomy and explicit clinical approval for high-impact clinical decisions. Rather than generating isolated outputs, agent systems can generate diagnostic or treatment plans, execute subtasks through controlled tool use, monitor outcomes, and adapt strategies based on feedback from the clinical setting. Recent studies have converged on a recurring set of functional components underlying such systems, often defined by 4 core components: planning, action, reflection, and memory (PARM) [11]. Planning refers to the decomposition of higher-level clinical goals into executable steps, action encompasses the execution of these steps through interaction with clinical information systems or decision support tools, reflection involves the evaluation of outcomes and system performance, and memory enables the storage and retrieval of contextual information over time [12,13]. In subsequent sections of this study, the 4 core components will be referred to collectively using the acronym PARM for clarity. Although these components have been defined across various branches of the literature, including multimodal AI, autonomous agents, and clinical informatics, they are often discussed in isolation or within domain-specific applications. Consequently, the field lacks a unified synthesis that positions medical AI agents within the broader evolution of CDSS and that explains how agent architectures expand upon, rather than replace, existing multimodal approaches [14]. This fragmentation makes it difficult for clinicians, researchers, and regulators to evaluate agent systems; compare designs; and reason about safety, accountability, and clinical value.

The aim of this Viewpoint is to synthesize current research on medical AI agents for clinical decision support by organizing the literature around the architectural dimensions of PARM. Rather than proposing a new standard or formal framework, this study uses these components as an analytical lens to examine how agent capabilities are incorporated into contemporary CDSSs. This Viewpoint aims to provide conceptual clarity and practical guidance for the responsible development and deployment of medical AI agents in clinical practice by tracing the transition from multimodal predictive systems to agent-based architectures, highlighting representative clinical applications, and discussing key safety and governance considerations.

Throughout this Viewpoint, PARM is used as an analytical lens to examine how agentic architectures extend traditional CDSSs. The purpose is not to provide an exhaustive review of all published studies but to offer a conceptual perspective on the emerging role of medical AI agents in clinical workflows.

## The Shift Toward Agentic Clinical Decision Support

Although multimodal AI enables the integration of medical imaging, clinical text, laboratory data, and physiological signals, many clinical applications remain limited to single-step inference. For example, a chest X-ray model may generate diagnostic predictions, while a clinical language model may summarize notes. These systems produce outputs but cannot autonomously pursue clinical goals across lengthy workflows.

Medical AI agents represent a qualitative shift. Instead of providing static predictions, agent-based systems iteratively plan sequences of actions, execute them through tool use, monitor outcomes, and adapt strategies based on feedback [9]. Recent studies have shown that LLMs can perform complex clinical tasks, access calculators and databases, interpret results, and refine their approaches through reflective reasoning [12,15]. This enables goal-directed behavior: an agent tasked with “optimize anticoagulation for this patient” can retrieve guidelines, assess bleeding risk, check for drug interactions, and recommend monitoring programs—tasks that previously required coordination between multiple independent systems or manual processes.

**Figure 1.**
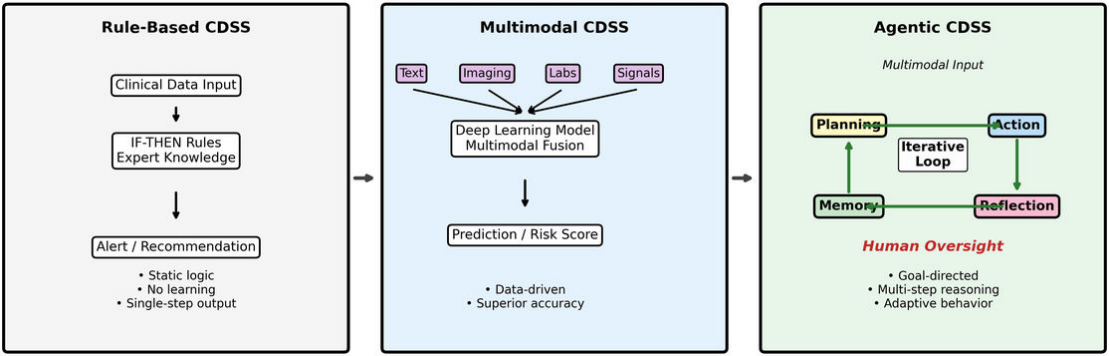
Evolution of clinical decision support systems (CDSSs) from traditional rule-based to agent-based architectures. Rule-based CDSSs rely on deterministic IF-THEN logic and expert-encoded information to generate alerts or recommendations from single data types, exhibiting static behavior with no learning or memory capabilities. Multimodal CDSSs integrate various data sources (clinical text, imaging, laboratory results, and physiological signals) through deep learning models to generate predictions or risk scores. Although multimodal fusion increases accuracy, these systems remain limited to single-step inference and reactive responses. Agent-based CDSSs extend multimodal capabilities by iteratively traversing the stages of planning (goal decomposition), action (tool use and execution), reflection (outcome evaluation), and memory (contextual retention). This PARM (Planning, Action, Reflection, and Memory)–based arrangement enables goal-directed, multistep reasoning and adaptive behavior. Human-supervised checkpoints provide a form of limited autonomy by requiring clinician approval for high-risk decisions. The shift from static prediction to iterative, adaptive assistance represents a qualitative change in clinical AI capabilities.

Figure 1 schematically illustrates the PARM architecture for an agentic CDSS with integrated clinical supervision.

On the basis of this evolutionary perspective, the following section examines the fundamental architectural components of PARM that enable agent behavior in CDSSs as interdependent functional elements.

## Key Architectural Components of Medical AI Agents

### Overview

Medical AI agents differ from traditional CDSSs through the integration of architectural components that enable goal-oriented, adaptive behavior in clinical workflows. In this section, PARM is considered a set of distinct yet interdependent elements, referred to as PARM components, that serve as an analytical lens rather than a prescriptive framework. Recent literature has revealed 4 interdependent components of central importance in the design of agent systems in health care. These components are not unique to medicine, but their applications and limitations are shaped by clinical uncertainty, safety requirements, and the need for human oversight. In this section, each component is examined in turn, with an emphasis on its role in clinical decision support. To provide a structured overview of these components and their roles in clinical decision support, Table 1 summarizes the functional goals, representational capabilities, and key considerations related to PARM in medical AI agents.

**Table 1.** Architectural components of medical AI^a^ agents in clinical decision support.

Components	Functional role in clinical decision support systems	Example capabilities	Key considerations	Technology enablers
Planning	Goal decomposition and task sequencing	Diagnostic pathways and care planning	Transparency and clinician alignment	Large language models and reasoning frameworks
Action	Tool-assisted execution	Electronic health record queries and guideline retrieval	Authorization and accountability	APIs^b^ and retrieval systems
Reflection	Outcome evaluation and performance monitoring	Error detection and performance drift monitoring	Auditability and traceability	Feedback loops and monitoring systems
Memory	Context retention over time	Longitudinal patient context	Privacy and governance	Vector databases and knowledge graphs

a AI: artificial intelligence.

b API: application programming interface.

Although these components can be defined individually, their clinical benefits emerge from their coordinated work within real-world workflows. Therefore, each component is discussed in more detail below, considering both its independent function and its interaction with the broader agent system.

### Planning

Planning enables medical AI agents to break down high-level clinical goals into executable sequences of action. Given an objective such as “Evaluate this patient for acute coronary syndrome,” an agent must guide subsequent steps by determining which diagnostic tests to order, in what order, and how to interpret the results. Planning systems may leverage clinical guidelines, task-specific protocols, and contextual patient data to create sequences of action that balance comprehensiveness and efficiency. Advanced planning frameworks can generate conditional plans that predict multiple possible outcomes and define appropriate responses for each [12]. This hybrid approach allows agents to remain flexible while adhering to domain-specific requirements.

However, the main implementation challenge is ensuring that automatically generated plans remain clinically sound, evidence-based, and appropriately constrained; that is, they should not exceed the agent’s defined scope of autonomy without explicit clinical approval. Overly aggressive or autonomous planning risks conflicting with clinician intent or patient preferences, highlighting the importance of transparency and controllability. Because of this uncertainty and these ethical considerations, planning components in medical AI agents are typically designed to support collaborative decision-making rather than independent execution.

### Action

Action encompasses the execution of planned steps through interaction with clinical information systems, decision support tools, or external information sources. Unlike traditional CDSSs, whose outputs are often limited to warnings or recommendations, agent-based systems can initiate controlled actions such as querying electronic health records, retrieving clinical guidelines, performing calculations, or generating structured reports.

Recent studies on tool-assisted reasoning have shown how AI systems can intertwine reasoning with action selection, allowing agents to select appropriate tools, interpret the returned information, and update subsequent steps accordingly [9]. In clinical settings, especially when interacting with live systems, such actions must be carefully limited to avoid unintended consequences. Consequently, many medical AI agents operate within predefined action domains and require explicit human approval for high-impact actions.

The distinction between recommendation and execution is critical. Although action-capable agents can streamline workflows and reduce cognitive load, their deployment must maintain clinician responsibility. Therefore, current applications focus on auxiliary actions, in which agents carry out preparatory or information-gathering tasks while final decisions and interventions remain under human control.

### Reflection

Reflection refers to an agent’s capacity to evaluate outcomes under clinical management, monitor its own behavior, and support iterative improvement. Reflection is particularly important in medical AI agents because performance failures often stem not from isolated prediction errors but from workflow-level issues such as premature shutdown, inappropriate tool use, or failure to report ambiguity. In practice, reflective functions can operate at three levels: (1) encounter-level controls (eg, identifying missing data, inconsistencies, or low-reliability recommendations requiring clinician review), (2) operational monitoring (eg, monitoring alert payload, override frequency, and abnormal action patterns), and (3) postdeployment surveillance (eg, detecting performance deviation and subgroup performance differences). In clinical settings, reflection is typically designed to support auditability and safety rather than autonomous self-modification. Continuous adaptation without oversight can reduce reproducibility and complicate accountability; therefore, reflective outputs should be logged, auditable, and linked to predefined update policies. This approach provides more secure integration into clinical workflows while preserving the authority of clinical physicians and traceable decision-making processes [13].

### Memory

Memory provides the structural foundation for continuity in agent-based CDSSs. By enabling the storage and retrieval of contextual information over time, it supports longitudinal reasoning and patient-specific adaptation. Memory in medical AI agents can encompass short-term context, such as the current clinical encounter, as well as longer-term representations, including previous decisions, outcomes, and relevant patient history.

Architecturally, memory systems can combine structured databases, vector-based retrieval mechanisms, and external information stores [16]. Recall-enhanced approaches allow agents to access current clinical information or institutional protocols without relying solely on static model parameters [10]. In patient-centered applications, memory supports more personalized and consistent decision support by enabling agents to be aware of evolving clinical trends.

At the same time, memory also raises significant concerns regarding privacy, data management, and bias [17]. Decisions about what information to store, for how long, and how to reuse it must comply with regulatory requirements and ethical standards. Therefore, the memory components in medical AI agents are closely intertwined with governance frameworks and organizational policies.

## Operationalizing Agentic Architectures in Clinical Decision Support

### Overview

The examples in this section are illustrative scenarios intended to show how agentic capabilities can be operationalized in CDSSs; they are not presented as validated end-to-end clinical systems unless explicitly supported by the cited literature. Although architectural components such as PARM define the conceptual foundation of medical AI agents, their clinical significance depends on how these capabilities are embodied in real-world decision support workflows. Operationalization requires aligning agent behavior with the temporal structure of clinical care, existing health information systems, and accountability frameworks governing medical decision-making processes. In this section, we highlight both opportunities and limitations by examining how agent architectures are implemented in key clinical decision support contexts [18,19].

### Diagnostic Decision Support

Diagnostic reasoning is an inherently iterative process involving hypothesis generation, targeted data collection, interpretation, and refinement [14]. An illustrative diagnostic AI agent might be directed by a command such as, “72-year-old patient with chest pain, elevated troponin, and nonspecific findings on ECG.” The agent plans differential diagnoses (eg, acute coronary syndrome, pulmonary embolism, and aortic dissection), retrieves relevant clinical guidelines, checks recent imaging and laboratory results, identifies missing data (eg, D-dimer status and previous cardiac history), and constructs a structured assessment with confidence estimates and suggested next steps [20]. Through reflection, the agent flags conflicting findings or low-reliability elements requiring clinical review. Major risks include premature diagnostic closure, anchoring bias, or failure to recognize rare but critical diagnoses. Monitoring mechanisms include mandatory clinical review before any diagnostic conclusion is communicated to patients, confidence thresholds that trigger automated amplification, and audit trails documenting the agent’s reasoning path. These trails are used for subsequent review and learning.

### Treatment Planning

In an illustrative scenario, treatment planning agents may extend beyond diagnosis, recommending therapeutic interventions tailored to individual patient contexts [21]. Given a confirmed diagnosis such as “newly diagnosed atrial fibrillation with moderate stroke risk,” the agent uses current guidelines (eg, Congestive heart failure, Hypertension, Diabetes, Stroke/Transient Ischemic Attack (TIA)/Thromboembolism, Vascular disease, Age, Sex category scoring and anticoagulation options), assesses contraindications based on medication history and laboratory values, considers patient-specific factors such as renal function and bleeding risk, and recommends treatment options in order of appropriateness. The agent can simulate expected outcomes under different scenarios, flag potential drug-drug interactions, and suggest monitoring protocols. Through its memory component, the agent remains aware of previous treatment responses and side effects. Risks include inappropriate dosage recommendations, failure to consider rare contraindications, or inadequate assessment of patient preferences. The review process includes mandatory clinical approval before any treatment recommendation reaches the patient, real-time alerts for high-risk recommendations (eg, anticoagulation in patients with recent bleeding), and documentation of the rationale for each decision for further review.

### Monitoring and Surveillance

In a hypothetical but clinically plausible workflow, a monitoring agent may track a patient’s condition over time, identifying clinically significant changes and triggering appropriate responses. For a patient receiving warfarin therapy, such an agent may continuously review incoming international normalized ratio values, compare them to target ranges, assess trends rather than isolated measurements, and identify factors that may explain deviations (eg, recent medication changes, missed doses, and dietary changes). When values fall outside acceptable limits, the agent generates alerts calibrated according to the level of urgency—immediate notification for critical values and scheduled review for borderline results. Such an agent may maintain longitudinal context through memory and recognize patterns, such as recurring subtherapeutic levels, that may indicate noncompliance. Key risks include alert fatigue from overreporting, critical changes missed due to improperly calibrated thresholds, or failure to report emergencies. Monitoring mechanisms include adjustable sensitivity parameters reviewed by clinical leadership, mandatory human consent prior to dose adjustments, and regular review of alert response times and outcomes [22].

### Workflow Integration

In all diagnostic, treatment, and monitoring applications, successful implementation requires seamless integration into existing clinical workflows. This includes compatibility with electronic health record systems, minimal disruption to established practices, and clear protocols for transitioning between automated processes and human decision-making. Implementation must consider varying levels of technical infrastructure, clinicians’ familiarity with AI systems, and organizational governance frameworks that define appropriate use boundaries [23,24].

## Discussion

### Safety, Governance, and Human Oversight

This study identified PARM as recurring functional components in medical AI agents and highlighted bounded autonomy, auditability, and workflow integration as central implementation themes. The capabilities that enable medical AI agents to achieve clinical goals in multistep workflows (PARM) bring governance challenges not found in traditional decision support systems. Static predictive models provide discrete outputs for human review; agent systems initiate sequences of actions that can take hours or days, access multiple data sources, and modify their behavior based on intermediate results. Ensuring patient safety and maintaining appropriate human authority require governance frameworks tailored to these different operational characteristics [25].

Bounded autonomy refers to the operational boundaries within which an agent can function independently and does not require explicit human authorization [26]. Boundaries must be specified across multiple dimensions: clinical scope (eg, which situations and which patient populations), authority to act (eg, information retrieval and order entry), and decision risk (eg, routine monitoring and critical interventions). A monitoring agent tracking vital signs postoperatively can autonomously retrieve laboratory results and assess trends but must consult a clinician before adjusting drug dosages or ordering imaging studies. Implementation typically requires the clear specification of permitted actions, definite stop points preventing unauthorized actions regardless of the agent’s recommendations, and clear escalation pathways when agents encounter situations outside their defined scope. Boundaries generally reflect not only clinical risk but also institutional capabilities, local practice patterns, and regulatory constraints. As agents demonstrate reliable performance in narrow scopes, boundaries can be gradually expanded through formal validation processes. In early deployment settings, a more restrictive operational scope with explicit clinician oversight may reduce safety and accountability risks [27,28].

**Figure 2.**
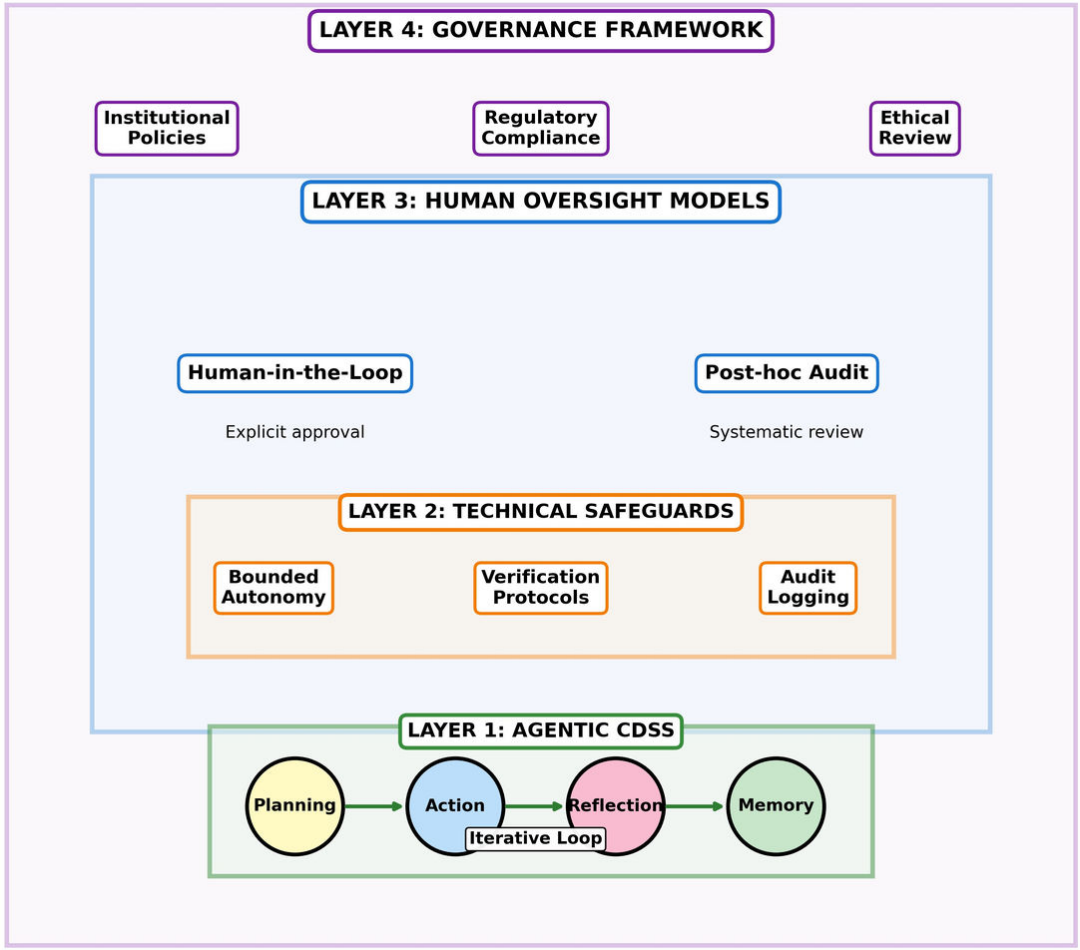
Multilayered human oversight framework for medical AI agents. The figure illustrates a multilayered defense-in-depth oversight architecture for agent-based clinical decision support systems (CDSSs). Agent behavior is primarily constrained by limited autonomy, technical security measures, and audit logs, while human oversight operates through clinician approval checkpoints, real-time audits, and postdeployment audit reviews. These layers are embedded within broader institutional, regulatory, and ethical governance structures, providing continuous oversight throughout the system life cycle.

Effective oversight operates across multiple temporal phases: predeployment validation, real-time monitoring during operation, and postoperative review of outcomes [29]. Predeployment validation extends beyond traditional model performance metrics to assess an agent’s behavior across anticipated clinical scenarios, including edge cases, ambiguous presentations, and situations requiring appropriate escalation. Validation should involve domain experts who evaluate not only the final recommendations but also the intermediate reasoning steps and calls to action. During the study, real-time monitoring tracks agent actions against expected patterns and flags anomalies such as excessive calls to action, unusual query patterns, or recommendations that deviate from established guidelines without clear justification. Confidence scores and uncertainty estimates should trigger mandatory human review when agents encounter unusual situations. Postdeployment monitoring collects performance data across patient encounters and identifies systematic errors, shifts in behavior over time, or performance differences among patient subgroups. Together, these temporal layers contribute to a multilayered defense, ensuring that failures of a control mechanism do not compromise patient safety. Figure 2 illustrates a multilayered human oversight framework for medical AI agents, where safety measures at the design stage, runtime oversight, and postdeployment management work concurrently to provide a deep-seated defense architecture that protects clinician authority while restricting agent behavior.

Complete auditability requires recording not only agent outputs but also the reasoning processes, data sources, and intermediate steps that led to those outputs [30]. Audit trails should record which guidelines were consulted, how patient-specific factors influenced the recommendations, which alternative options were evaluated and rejected, and when human review was initiated. This transparency serves multiple functions: it allows clinicians to understand and validate the reasoning of agents, supports quality improvement by identifying recurring errors or suboptimal patterns, and ensures accountability when outcomes are negative. Most importantly, accountability remains with human clinicians, not AI systems, even when decision support becomes more agentic and workflow integrated [31]. Agents function as assistive tools supporting the clinical decision-making process; ultimate responsibility for patient care rests with licensed professionals who have the authority to accept, modify, or reject agent recommendations. Documentation systems should clearly state which actions were initiated by agents, which were approved by clinicians, and the justification for any deviations from agent recommendations. This ensures the effective use of agent support while preserving established medical accountability frameworks.

Evaluating agent systems requires measures that go beyond predictive accuracy to assess task completion, behavioral appropriateness, and modes of failure [29]. Task reliability measures whether agents successfully complete clinical workflows from start to finish, not just whether individual predictions are correct. An agent might correctly diagnose a condition but fail to order appropriate validation tests or inform the relevant clinician; this represents a task failure despite diagnostic accuracy. Constraint compliance monitoring tracks whether agents operate within defined limits and flags violations even if the results are acceptable. An agent ordering imaging studies outside its authorized scope raises managerial concerns regardless of whether the imaging is clinically appropriate. Recovery and decay models assess how agents respond to errors, ambiguous inputs, or missing data, determining whether they fail by escalating appropriately or produce unreliable outputs without signaling the ambiguity. Evaluation frameworks are expected to highlight not only what agents do correctly but also how they behave when faced with conditions outside those encountered during training, as real-world deployments inevitably involve scenarios not represented in development datasets.

As medical AI capabilities evolve toward agentic architectures, governance frameworks must evolve in parallel. Using PARM as an analytical lens may help developers, clinicians, and regulators design agentic systems that balance clinical benefit with appropriate constraints, transparency, and human authority. Future work should establish standardized evaluation protocols for agentic CDSSs, develop consensus guidelines for limited autonomy in clinical settings, and explore how human-agent collaboration patterns evolve as agents assume greater workflow responsibilities while maintaining patient safety and professional accountability.

### Open Challenges and Future Directions

Despite their promise, medical AI agents face significant challenges that must be addressed before they can move into widespread clinical use [32]. A fundamental technical challenge involves managing context windows across lengthy patient encounters. Although current LLMs exhibit impressive reasoning capabilities, their safe and reliable clinical use remains an active area of investigation [33]. Agents must integrate information that may arrive asynchronously (eg, laboratory results arriving hours after the initial assessment, imaging reports from external facilities, or expert consultations occurring days later) while maintaining diagnostic consistency and avoiding contradictory recommendations. The evaluation challenge extends beyond traditional benchmarks. Current benchmarks primarily evaluate isolated predictions on static datasets and fail to capture the performance of agents in multistep clinical workflows where intermediate decisions influence subsequent actions [34]. Developing assessment frameworks that measure end-to-end task completion, appropriate escalation behavior, and incremental deterioration under uncertainty represents an important research priority. Such frameworks should also address distributional drift, as agents trained with data from specific institutions or populations may exhibit unexpected failures when deployed in different clinical contexts [35].

Regulatory frameworks currently lack clarity regarding agent systems [36]. Current medical device regulations address static algorithms that produce discrete outputs; agent systems that initiate sequences of actions, access multiple data sources, and modify their behavior based on intermediate outcomes challenge traditional concepts of validation and approval. Determining appropriate regulatory pathways (ie, whether agents should be validated as complete systems or whether their component capabilities should be independently validated) remains an open question requiring collaboration among regulators, developers, and clinical stakeholders.

Long-term opportunities include developing agents that can explain their reasoning in clinically meaningful terms, moving beyond attention maps and attention weights to generate medically informed rationalizations. Furthermore, enabling agents to work effectively in resource-constrained environments where access to comprehensive electronic health records or high-bandwidth connectivity may be limited could extend their benefits beyond well-resourced health care systems [37,38]. As these technologies mature, research should also address how human-agent collaboration models evolve and ensure that agents support, rather than undermine, clinical expertise.

### Study Limitations

This conceptual perspective has several limitations. PARM, as an analytical lens in this paper, has not been empirically validated with controlled trials comparing agent-based and traditional CDSSs. The clinical case examples presented are illustrative examples rather than systems currently used in clinical practice, and their real-world performance must be determined through prospective trials. Our discussion emphasizes technical and managerial considerations without conducting a comprehensive analysis of implementation costs, organizational change management, or clinician training requirements that would significantly impact adoption. Additionally, the rapidly evolving nature of LLM capabilities means that certain technical limitations discussed may be addressed with newer model architectures, but fundamental managerial challenges are likely to persist regardless of the underlying technology.

### Conclusions

Medical AI agents extend CDSSs beyond isolated predictions, broadening them toward workflow-driven assistance in which systems can plan tasks, execute controlled actions, evaluate performance, and maintain context over time. Using PARM as an analytical lens, this Viewpoint clarifies how agent capabilities can be made functional across diagnostic reasoning, treatment planning, and longitudinal monitoring while remaining constrained by clinician oversight and institutional governance. From a computational standpoint, the practical impact of agent-based CDSSs will depend on interoperability with clinical information systems; transparent record and audit trails; and assessment approaches that capture end-to-end task reliability, constraint compliance, escalation behavior, and security under deployment shift. Future work should prioritize rigorous implementation studies, standardized reporting of agent behavior, and governance models that support responsible deployment without displacing clinical responsibility. Properly designed medical AI agents can reduce cognitive load and improve the coordination of information and tasks while preserving human judgment as the ultimate authority in patient care.

## Abbreviations

CDSS: clinical decision support system
LLM: large language model
PARM: planning, action, reflection, and memory
